# Movement patterns of an arboreal marsupial at the edge of its range: a case study of the koala

**DOI:** 10.1186/2051-3933-1-8

**Published:** 2013-09-12

**Authors:** Nicole Davies, Galina Gramotnev, Leonie Seabrook, Adrian Bradley, Greg Baxter, Jonathan Rhodes, Daniel Lunney, Clive McAlpine

**Affiliations:** School of Biomedical Sciences, The University of Queensland, St Lucia, Queensland 4072 Australia; The University of Queensland, Landscape Ecology and Conservation Group, School of Geography, Planning and Environmental Management, St Lucia, Queensland 4072 Australia; The University of Queensland, Environmental Decisions Group, St Lucia, Queensland 4072 Australia; Office of Environment and Heritage NSW, PO Box 1967, Hurstville, New South Wales 2220 Australia; School of Veterinary and Life Sciences, Murdoch University, Perth, Western Australia

**Keywords:** Koala, Semi-arid, Resource selection, Spatial distribution, Australia, Home range

## Abstract

**Background:**

Conservation strategies derived from research carried out in one part of the range of a widely distributed species and then uniformly applied over multiple regions risk being ineffective due to regional variations in species-habitat relationships. This is particularly true at the edge of the range where information on animal movements and resource selection is often limited. Here, we investigate home range size, movement patterns and resource selection of koalas *Phascolarctos cinereus* in the semi-arid and arid landscapes of southwest Queensland, Australia. We placed collars with GPS units on 21 koalas in three biogeographic regions. Home range sizes, resource selection and movement patterns were examined across the three regions.

**Results:**

Habitat selectivity was highest at the more arid, western edge of the koala’s range with their occupancy restricted to riparian/drainage line habitats, while the more easterly koalas displayed more variability in habitat use. There was no significant difference between home range sizes of koalas at the western edge of the range compared to the more easterly koalas. Instead, variability in home range size was attributed to spatial variations in habitat quality or the availability of a key resource, with a strong influence of rainfall and the presence of freestanding water on the home range size of koalas. Within a 580 m spatial range, movement patterns of male and female paths showed a tortuous trend, consistent with foraging behavior. Beyond this spatial range, male paths showed a trend to more linear patterns, representing a transition of movement behavior from foraging to breeding and dispersal.

**Conclusions:**

The difference in home range movement patterns and resource use among the different koala populations shows that behavior changes with proximity to the arid edge of the koala’s range. Changes in home range size and resource use near the range edge highlight the importance of further range-edge studies for informing effective koala conservation and management actions, especially when developing species-specific adaptation responses to climate change.

**Electronic supplementary material:**

The online version of this article (doi:10.1186/2051-3933-1-8) contains supplementary material, which is available to authorized users.

## Background

Widely distributed species have natural geographic ranges extending over multiple biogeographic regions. Conservation strategies derived from research carried out in a limited part of a species’ range, then uniformly applied over multiple regions, risk being ineffective for those species that occupy different habitat types and climatic zones across their range [1, 2]. Despite the potential importance of this problem for species’ conservation, currently there is limited understanding of regional variation in species-habitat relationships within broad geographic ranges [1].

This problem is particularly important where knowledge about the movement patterns of individuals at the boundary of the species’ geographic range is limited [3], although there is evidence that the scale of movements at the boundary is greater than at the center [4–6]. For example, the largest home ranges of raccoons were found at the northern (coldest) edge of their distribution, which was considered to be a function of sparse resources [5]. Further, habitat selectivity can be predicted to be higher in landscapes located at the edge of a geographic range because high-quality habitat resources are scarcer [7]. However, food is not always the limiting resource [8]. Low population densities and shelter availability can contribute to larger home range sizes to meet physiological or breeding requirements [6].

Distributional changes are occurring at the boundaries of species’ geographic ranges as a consequence of climate change [9]. This includes boundary expansions on the leading edge of a range and contraction at the trailing edge, with climate change leading to increasingly fragmented populations on the trailing edge [9]. Some populations may survive in refugia, while others will face local extinction from extreme climatic events [10]. It has been shown that trailing-edge populations can be critical to the long-term survival of species because they may contain individuals that can adapt to changing climatic conditions [11–13]. Investigating movement patterns and resource selection at the trailing edge of a widely distributed species’ boundary, and how these vary across an increasingly arid climate gradient, will allow us to improve our understanding and management of animal-habitat relationships by identifying areas that will provide suitable habitat in a changing climate as well as facilitate decisions to prevent further contractions in a species’ distribution.

The movement ecology framework provides a relatively complete view of the basic processes involved in individual movement [14, 15]. It depicts the interplay among four mechanistic components of organismal movement: three components are related to the focal individual - the internal state (why move), motion capacity (how to move) and navigation capacity (when and where to move) - and the fourth component refers to the external (environmental) factors affecting movement [15]. The internal state (including an organism’s physiological state and its short-term motivation in relation to its long-term “goals” – e.g. reproduction, maintenance, survival), motion and navigation factors can be modified by external environmental factors, including the landscape, meteorological and other physical factors, the distribution of resources and different environmental conditions, other organisms (including conspecifics and interspecific – e.g. mates, competitors, predators), and coordinated group movements [14, 15].

The koala is an arboreal marsupial folivore, which feeds almost exclusively on a limited range of tree species of the genera *Eucalyptus*, *Corymbia* and *Angophora*. It is widely distributed, with its range extending across 30 bioregions from tropical Queensland to temperate Victoria and South Australia. Southern and eastern koala populations have been relatively well studied compared with koalas in semi-arid western Queensland. Koala home ranges in more mesic regions vary between 1 ha to 300 ha [16, 17]. In Queensland, home range sizes vary from 5.6 ha to 296 ha in central Queensland [16]; 0.6 ha to 39.9 ha at St Bees Island on the central Queensland coast [18]; and 5.3 ha and 91.4 ha in agricultural landscapes in southeast Queensland [19]. However, the size of the home range at the western limits of its geographic range, and how this varies across an increasingly arid climate gradient, has not been examined.

Climate variability and nutrients are the primary drivers of ecological processes in arid and semi-arid landscapes. Both are temporally and spatially variable, generating heterogeneous ecosystems [20]. Munks *et al.*[21] and Gordon *et al.*[22] point out that water availability (including leaf moisture) is a primary factor defining preferred habitat for arboreal marsupials, such as the koala, in semi-arid regions. In these regions, koalas are most commonly found in riverine habitats [21–25], although they do utilize other habitats [16, 26, 27]. Munks *et al.*[21] found a strong relationship between the density of koalas in semi-arid northern Queensland, proximity to surface water bodies and the leaf moisture of preferred *Eucalyptus species*. Using indirect methods (koala pellet, i.e. dung surveys), Seabrook *et al.* and Smith *et al.*[23, 25] identified river red gums *Eucalyptus camaldulensis* in riparian habitats as the primary resource for koalas in southwestern Queensland.

Investigating home range movement patterns and resource selection [28], at the scale of individual animals, allows us to characterize selection at fine spatial scales, improve our understanding of animal-habitat relationships, and facilitate better management decisions [29]. This is especially important for species vulnerable to climate change at the margins of their distribution. This requires monitoring of individual movements to detect fine-scale patterns of resource use. Understanding this relationship is especially important for tree-dependent species because specialization can restrict their access to essential resources [30].

The aim of this study was to investigate home range sizes, movement patterns and resource use of koalas in semi-arid landscapes. The hypotheses tested were that the home range size of koalas at the trailing edge of their range distribution would be larger, movement patterns would be more linear, and they would have higher habitat selectivity than populations further east towards the core of their range. To test these hypotheses, we radio-tracked 21 koalas, with the addition of GPS units, across three different biogeographic regions (bioregions) located at varying distances from the leading edge of the koala’s range. The study was located in southwestern Queensland, Australia (geographic extent ~ 200,000 km^2^), where koalas are at the western limits of their geographic range and form a trailing-edge population [23].

## Results

### Home range sizes

A total of 21 koalas (10 females, 11 males) were tracked between August 2010 and November 2011 (Additional file 1). GPS fixes were recorded at four-hour intervals. An asymptote for the curve for the fixed kernel home range was estimated, with a mean of 224 and standard deviation SD = 152.9 (median of 155) locations per koala to reach asymptote. Due to equipment failures and other technical problems, the GPS units of four koalas either recorded no data or insufficient data (between 0-66 fixes and 0-14 days of data for these four koalas) to warrant inclusion. The home ranges of three male koalas (one from the Mulga Lands bioregion and two from the Brigalow Belt South bioregion) did not reach an asymptote, but were included in home range analyses because reaching an asymptote was not prerequisite for inclusion (inclusion: Kruskal-Wallis: *P* = 0.0920; exclusion: Kruskal-Wallis: *P* = 0.1548) (see Approach and limitations below). We calculated home range size for 17 koalas (8 male, 9 females). The mean 95% fixed kernel home-range size for the entire study area was 78.1 ha (± 33.1 ha) (Table 1). The Mulga Lands home ranges were larger than those on the Mitchell Grass Downs and Brigalow Belt South, and this difference was significant at the 0.09 level (Table 1) (home range maps for each koala are in Additional file 2). Fixed kernel core areas for the Mulga Lands were larger than those on the Mitchell Grass Downs and Brigalow Belt South bioregions and this difference was significant at the 0.09 level) (Table 1).

**Table 1:** **Home range sizes**

Bioregion	n	Fixed kernel: 95%	Fixed kernel: core
Mulga Lands	6	169.5 ± 85.1	45.1 ± 19.2
Mitchell Grass Downs	4	20.7 ± 5.7	6.1 ± 1.6
Brigalow Belt South	7	32.9 ± 8.8	9.8 ± 2.6
**Kruskal-Wallis test**	***P*** **= 0.0920**		***P*** **= 0.0900**

Mean and standard error (ha) for the fixed kernel 95% home range and core areas in each bioregion. Results of the Kruskal-Wallis tests comparing home ranges of the three bioregions.

### Influence of environmental variables on home range size

**Figure 1:**
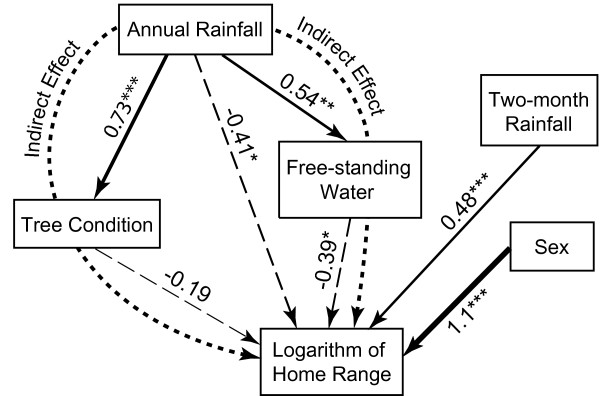
**Structural equation model diagram.** The structural equation model diagram with the identified (by the solid and dashed arrows) direct dependences between the variables (shown in the boxes). The numbers next to the arrows show the corresponding regression coefficients between the respective variables in the boxes. The asterisks indicate the levels of significance for the respective regression coefficients: (*) *p* < 0.05; (**) *p* < 0.01; (***) *p* < 0.001; the absence of the asterisks means statistical insignificance. The direction and thickness of the arrows indicate the direction (causality) of the mutual impact of the variables and the approximate values of the regression coefficients, respectively. The dashed arrows indicate the negative regression coefficients. The dotted arrows show the indirect effects of annual rainfall on home range.

The outcome of structural equation modeling (SEM) is shown in Figure 1, with the direct causal influences of the independent variables on log home range and on each other identified by arrows, where the pairs of variables displaying mutual correlations had *p*-values < 0.1 (Additional file 3). The log home range is used here because the original home range data were logarithmically transformed to achieve a normal distribution (see the Methods section and Statistical Analyses sub-section below). The nitrogen variable did not show significant direct or indirect effects.

The outcome of the SEM shows annual rainfall variable has large direct impacts on log home range, tree condition, and the amount of the freestanding water. Annual rainfall improves tree condition and increases the amount of available freestanding water, but decreases the size of the log home ranges (Figure 1). There is an indirect effect between annual rainfall and log home range size (*p* = 0.031). This indirect effect has two different pathways – through the mediator variables of tree condition and of freestanding water (Figure 1). The total effect of annual rainfall on log home range is ≈ – 0.77 (*p* < 0.001). The indirect effect of annual rainfall on log home range is ~ 46% of the total impact of the annual rainfall on log home range, with ~ 28% coming through freestanding water and ~ 18% coming through tree condition.

In our SEM model, the model *p*-value is equal to = 0.46 (> 0.1), the comparative fit index and Tucker-Lewis index were both equal to 1 (> 0.95), standardized residuals root mean square were equal to 0.089, and *R*^2^ ≈ 87%, which indicates the overall good model fit [31], with ~ 87% of the variance of the log home range variable explained by the considered model. Therefore, most of the factors affecting the size of the koala’s home range were taken into account by this model, and only ~13% of the variation of the koalas’ log home range was related to unmeasured variables.

The SEM model shows that tree condition works as a mediator between annual rainfall and log home range with no significant impact on its own. Therefore, the subsequent multiple regression analysis excluded tree condition as an independent variable.

### Effect of rainfall and sex on home range size

**Figure 2:**
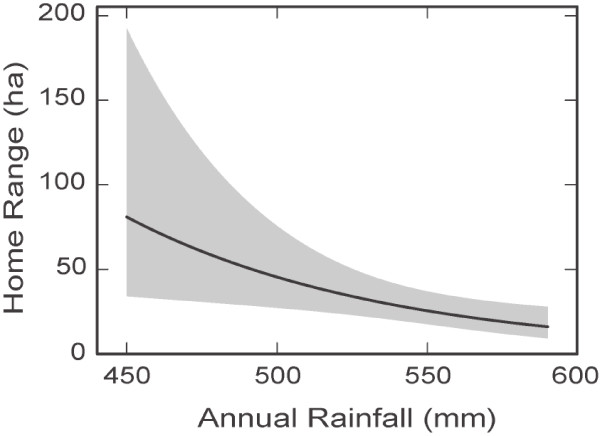
**The dependence of the home range size on annual rainfall.** The grey band shows the 95% prediction interval.

To investigate possible interactions and relationships between the variables, we used a multiple regression model. The multiple regression analysis was conducted on the set of the significant variables identified by SEM (annual rainfall, two-month rainfall, sex, and freestanding water) and their mutual interactions. Only annual rainfall, freestanding water, and the interaction term between sex and two-month rainfall appear to have significant effects on the koalas’ log home range. In the presence of this interaction, the direct effects of sex and two-month rainfall on log home range are not statistically significant, although the interaction term has the largest effect size, being equal to 0.29 (Table 2). Here, effect size is defined as a fraction (proportion) of variance that is attributed to a particular independent variable. This outcome shows that male and female koalas react differently to habitat changes as a result of rainfall in the preceding two months. Freestanding water and annual rainfall (Figure 2) had significant negative effects on home range size. The average home range size decreased from ~ 80 ha where annual rainfall was 450 mm per annum to ~ 18 ha where annual rainfall was 580 mm per annum.

**Table 2:** **Multiple regression**

Variable	Effect size	Regression coefficient	Standard error	P value
Annual rainfall	0.082	– 0.012	0.004	0.021
Two-month rainfall × Sex	0.29	0.014	0.003	0.001
Freestanding water	0.20	– 1.29	0.34	0.002

Multiple regression of koala log home range and explanatory variables.

**Figure 3:**
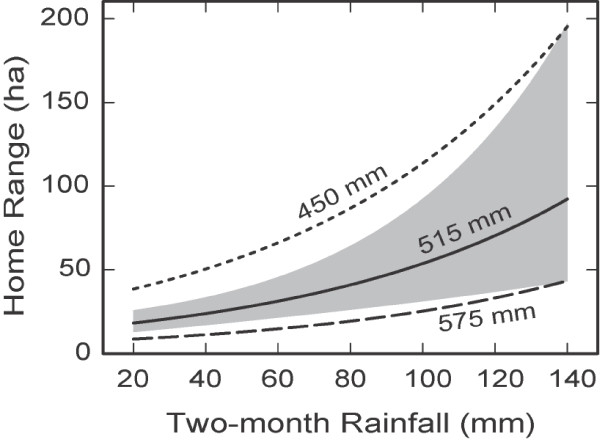
**Home range size (males) dependences on two-month rainfall for average, minimum and maximum annual rainfall.** The dependences of home range size for male koalas on two-month rainfall for the average annual rainfall of 515 mm (solid curve), for the minimum annual rainfall of 450 mm (dotted curve), and for the maximum annual rainfall of 575 mm (dashed curve) in the presence of freestanding water. The statistical contrasts (differences) between these three curves are significant (with *p* < 0.01). The grey band shows the 95% prediction interval for the average annual rainfall. No statistically significant dependence of home range size on two-month rainfall was found for female koalas.

The effect of two-month rainfall (Figure 3) differs from that of the annual rainfall (Figure 2). While an increase of annual rainfall causes reduction of home ranges for both males and females, an increase of short-term rainfall has the opposite effect on male koalas and does not have an effect on females. Decreasing annual rainfall causes a significant increase in mobility of male koalas as a result of increased two-month rainfall (compare the dashed, solid and dotted curves in Figure 3). The increase in home range size for male koalas with increasing two-month rainfall can be large – for example, from ~ 40 ha to ~ 200 ha where two-month rainfall increases from ~ 20 to ~ 140 mm under the condition of the minimum annual rainfall of 450 mm (Figure 3).

Model averaging showed that the variables/interaction terms had a similar significance in the calculated average model as in the multiple regression model with interaction: freestanding water (*p* = 0.005), annual rainfall (*p* = 0.07), and the interaction term between the sex variable and two-month rainfall (*p* = 0.049). There were no differences detected for all other variables/interaction terms (*p* > 0.05). In addition, the normality of the model residuals has been confirmed using the Q-Q-plots and Shapiro-Wilk test (*p* = 0.4). The goodness of fit test showed a good fit for the model: AIC (the Akaike information criterion) ~ 30.0, the residuals root mean square ~ 0.52, and the value of the coefficient of determination *R*^2^ adjusted for the considered sample size is ~ 81% (i.e., ~ 81% of the variance of the log home range variable can be explained by the considered model).

### Movement patterns and resource use

**Figure 4:**
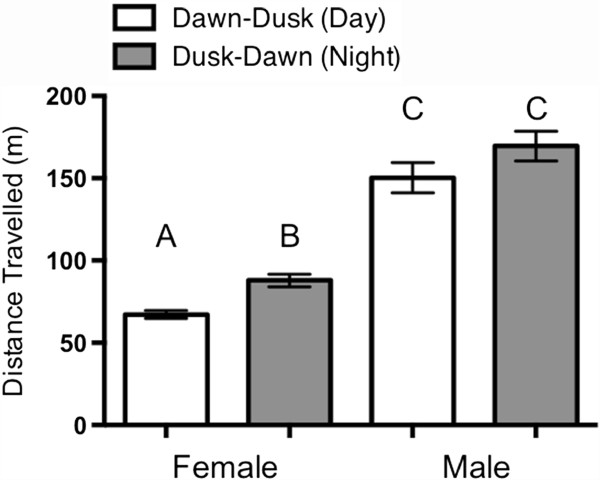
**Diurnal and nocturnal movement patterns.** Mean (± standard error) diurnal and nocturnal movement patterns of males and females. **A**, **B** and **C** identify that significant differences exist from the *post hoc* test: all pairs of combinations were significantly different except for the male day and night comparison.

**Figure 5:**
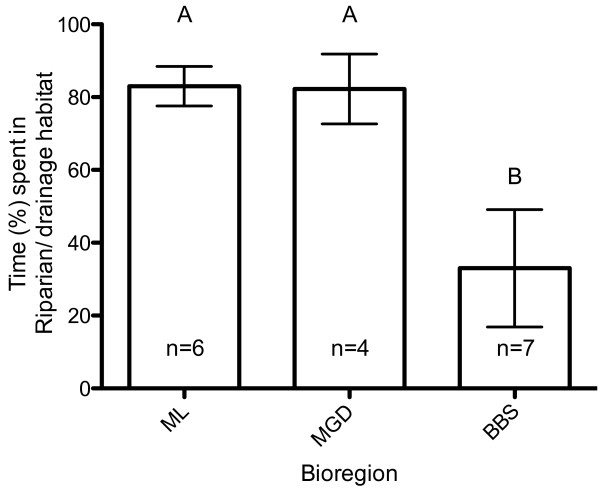
**Time spent along drainage/riverine habitat.** Mean (± standard error) time spent (%) along drainage/riverine habitat (i.e. time spent on the creek/drainage-line rather than in off, non-riverine habitats) for koalas within the Mitchell Grass Downs (MGD), Mulga Lands (ML) and Brigalow Belt South (BBS) bioregions. **A** and **B** identify significant differences from the *post hoc* test.

Males (n = 8) travelled further both by day and night than females (n = 9) (one-way ANOVA: P < 0.0001) (Figure 4). The koalas in the western edge of the geographic range spent on average significantly more time (Mulga Lands 1574 ± 313.0 hours; Mitchell Grass Downs 1739 ± 510.8 hours) within riparian and drainage line habitat compared to the koalas in the more eastern Brigalow Belt South bioregion (186 ± 97.3 hours) (Figure 5) (one-way ANOVA, *P* = 0.017, *F*_2, 14_ = 5.526). There was no difference between mean tortuosity (Fractal dimension (D) mean) of paths between the three bioregions (Table 3) (ANOVA: P > 0.05 respectively).

**Table 3:** **Fractal D**

Variable	Mean fractal D (± SE)
Mulga Lands	1.355 ± 0.06103
Brigalow Belt South	1.446 ± 0.07625
Mitchell Grass Downs	1.416 ± 0.07625

Mean tortuosity of paths for males, females and each bioregion.

**Figure 6:**
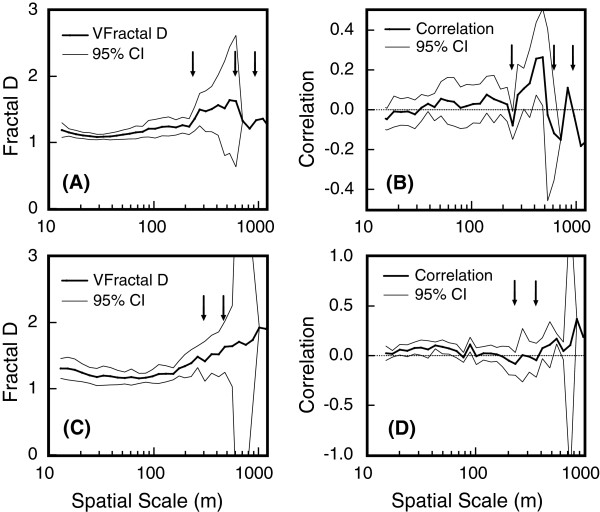
**Plots of fractal D and correlation in tortuosity of successive path segments of male and female koalas.** **(A)** Mean fractal D, and **(B)** correlation in tortuosity of successive path segments with corresponding 95% confidence intervals for male koalas (n = 8) at varying spatial scales. **(C)** Mean fractal D, and **(D)** correlation in tortuosity of successive path segments with corresponding 95% confidence intervals for female koalas (n = 9) at varying spatial scales. Arrows represent point of inflection on plots of mean D (**(A)** and **(C)**). Dotted lines represent correlation = 0, and arrows represent drops in correlation below zero on plots of correlation in tortuosity of successive path segments (**(B)** and **(D)**). Movement patterns of male koalas showed three changes in movement patterns, at approximately 240, 610 and 950 m (**(A)** and **(B)**). Movement patterns of female koalas showed two changes in movement patterns, at approximately 230 – 260 m (similar to males) and the second at path lengths of 360 - 400 m (**(C)** and **(D)**).

Plots of fractal dimension (D) versus spatial scale (Figure 6) revealed that tortuosity of male movement paths increased with increasing scale, except at the larger scales (> 580 m) where plots of D and correlation exhibited a discontinuity against spatial scale (Figure 6A). Males showed three changes in movement patterns at 240, 610 and 950 m (Figure 6A). The drop in correlations near these path lengths (Figure 6B) indicate that perceived patch size was within this range. Small-scale movement patterns between 230 and 580 m were more tortuous than at shorter distances (with a concomitant rise in D and a generally positive correlation) as the koalas were likely foraging around areas (Figure 6A). Above this threshold, the movement paths became more linear again as the koalas were likely traversing either between patches or in search of mates (the D decreased sharply).

For females, tortuosity also increased with increasing scale, however, there was no discontinuity against spatial scale as observed for males at the 580 m mark (Figure 6C). Instead, females showed two changes in movement patterns, one at 230 – 260 m (similar to males) and the other at 360 - 400 m (Figure 6C and D). Drops in correlations at these path lengths indicate that perceived patch size was within this range. Overall, it is likely that the perceived patch size for both males and females is covered by a direct path length of between 230–580 m, and the trend to more linear movement patterns of males past this threshold reflects a transition of movement behavior from foraging to breeding or dispersal.

The examination of the GPS point-by-point movement of the koalas shows a pattern of to and fro movement within a patch (with patch length up to approximately 580 m), then travel in a linear path to the next patch (where paths are again very torturous within the patch). These observations support the perceived patch size, identified from the plots of D and correlation in tortuosity, of successive path segments. The long path segments (i.e. > 580 m) of male koalas are usually followed by more path segments in the same general direction (and not tortuous) and suggest that these males were not foraging at these times. This further supports a trend to more linear movement patterns reflecting a transition of movement behavior from foraging to breeding as suggested by the plots of D and correlation in tortuosity for males.

We used multiple regression to quantify the effect of the explanatory environmental variables on movement patterns (the overall distance travelled and fractal dimension). The mean fractal dimension variable showed significant dependence on annual rainfall, sex, and 2 month rainfall. As with log home range, the two latter variables are significant through their interaction term. The model averaging procedure showed significance for the same variables of annual rainfall and the interaction term between sex and 2 month rainfall. Mean fractal dimension for koala movements increase with increasing annual rainfall, which corresponds to decreasing home range area in the above analysis. For male koalas, mean fractal dimension decreases with increasing 2 month rainfall, which is again consistent with the previously obtained outcomes for the log home range variable (i.e., home range increases). However, the percentage of the explained variance R^2^ ≈ 54% is significantly smaller than for the regression with log home range as the dependent variable for which R^2^ ≈ 81% (see above).

The multiple regression analysis with the log distance variable, instead of log home range (the distance variable required logarithmic transformation to achieve its normality), shows that sex, and 2 month rainfall are significant independent variables. The model averaging procedure did not show significance better than 5% for any of the independent variables. The percentage of the explained variance was R^2^ ≈ 59%, compared to R^2^ ≈ 81% for the model with the log home range dependent variable.

As a result, the home range variable appears to be the most appropriate choice, as the dependent variable, in the multiple regression analysis primarily because it corresponds to the largest percentage of the explained variance. In addition, the SEM analysis for the mean fractal dimension and log distance variables instead of the log home range variable yielded R^2^ ≈ 78% and root mean square error of 13.8% (for the fractal dimension variable) and R^2^ ≈ 86.5% and root mean square error of 11% (for the log distance variable), compared to R^2^ ≈ 87% and root mean square error of 8.9% for the log home range variable. This result further confirms the suitability of the log home range as the best choice as the dependent variable.

## Discussion

An important prerequisite for the conservation and management of forest-dependent mammals is a sound understanding of how a species utilizes its habitat within different parts of its geographic range [2]. This study addressed the hypothesis that the home range size of koalas at the trailing edge of their range would be larger, movement patterns would be more linear, and they would have higher habitat selectivity than populations further east towards the core of their range. We found that koala home ranges were mainly influenced by rainfall (both annual and short term, i.e. two-month previous), the presence of freestanding water and whether the koala was male or female. Riparian habitat use was higher at the edge of the range, with koalas in the western region being mainly restricted to riparian habitats, while the eastern koalas displayed more variability in habitat use (spending less time in riparian habitats compared to their western counterparts). The differences in home range in relation to rainfall and water availability, and in resource use among the different koala populations, support the idea that animals living near the arid extremities of their range have to cope with lower resource quality and higher environmental stresses.

Evidence from studies in Europe, Canada and Australia indicates that space use and movement distances are greater in marginal habitats at the edge of the species’ range, with results showing correlations between home range size and resource abundance and population densities [4–6]. Results from this study are in accord with the other studies and show that this trend also occurs at the semi-arid, trailing edge of a species’ distribution with rainfall and freestanding water resources driving variation in home range sizes. Furthermore, the Mulga Lands bioregion, at the western, more arid edge of the koala’s distribution, had the largest home ranges, which were among the largest recorded anywhere [16, 18, 19].

A study of habitat use by prairie dogs in northern Mexico showed a higher degree of habitat selectivity in landscapes at the edge of their geographic range because high-quality resources were scarce [7]. Results from this study indicate that the western koalas in the Mulga Lands and Mitchell Grass Downs bioregions spent the majority of their time in drainage line habitats, whereas the more eastern koalas of the Brigalow Belt South bioregion displayed more variability in patterns of habitat use, utilizing either riparian or non-riparian habitat. Foliar moisture supplies most of the water requirements for koalas [21]. It follows that the leaf moisture content in trees within riparian habitats would be higher, hence increasing habitat quality. In dry environments, or during drought, it has been proposed that leaf moisture rather than leaf nutrients influences tree selection by koalas [21, 26]. In southwestern Queensland, it has been shown that the leaf moisture content and total phenolics were higher in *E. camaldulensis* in riparian habitats than in *E. populnea* which occurs in non-riparian habitats [32]. Understanding habitat selection and its spatial and temporal variability is particularly important for tree-dependent species because specialization on forest or woodland resources, such as riparian habitats, can highly restrict the movements and dispersal capacity of such species [30].

Phillips and Callaghan [33] and Rhodes [34] postulated that variations in home range sizes of koalas reflect habitat quality, whereby a sparsely distributed food resource dictates a requirement for larger home ranges. Although foliar moisture supplies most of the koala’s water requirements [21], animals have often been observed drinking from, and sitting in, waterholes during the summer months and during heatwaves (personal observation from local landowners; [27]). In this study, the observed reduction of koala home ranges with increasing annual rainfall is likely related to a greater availability of freestanding water and a greater abundance and/or quality of food resources (e.g. higher leaf moisture, better leaf coverage and better tree condition in areas that receive higher annual rainfall). Therefore, the greater abundance and/or quality of food and water resources in more mesic areas reduce the need for frequent and extensive movement to find resources.

Changes in environmental conditions can affect population dynamics, leading to populations expanding and contracting as conditions fluctuate [12, 35, 36]. Furthermore, time lags can occur between rainfall events and the subsequent response by vegetation [37–39]. Koalas collared in May 2011 experienced higher than average rainfall for the previous two months (e.g. at Charleville, rainfall in March 2011 was 189 mm compared to the March average of 60 mm (1942-2012) [40]). In areas with low annual rainfall, there was a significant increase in mobility of male koalas as a result of increased two-month rainfall. A possible explanation is that koalas are dispersing into less optimal habitat following high rainfall over the previous two months. This would reflect opportunistic use of trees that, in drier times, do not provide sufficient resources, but it may also reflect breeding or dispersal movements, especially since rainfall in the previous two months predominantly affected male rather than female koalas, and a male-biased dispersal has been observed in a number of studies [41, 42]. Previous studies have found that, although largely solitary, koalas occupy reasonably well-defined home ranges and both male and female home ranges generally overlap [16, 19, 41, 43].

The tortuosity of successive path segments of male and female koalas shows two distinct patterns. Over shorter distances (<580 m) both male and female movements show a trend to more non-linear (tortuous) movement patterns, which are likely to represent foraging behavior. Over greater distances (>580 m), male movements show a trend to more linear (less tortuous) patterns. These are likely to represent a transition of movement behavior from foraging to breeding and dispersal. Also, the mean fractal dimension of males showed a trend to more linear movement patterns with increasing short-term rainfall. Mean daily distances travelled by males were also larger than for females. This provides support that the larger, more linear distances travelled by males are related to breeding or dispersal. Male-biased dispersal has been observed in a number of studies [41, 42], and male home range sizes increased with short-term increases in rainfall. This suggests that in more arid landscapes, male koalas used the intermittent window of opportunity for mating and dispersal in response to significant short-term improvement in conditions related to increased short-term rainfall.

By using direct monitoring of individuals, we were able to identify the importance of freestanding water sources, particularly dams, to koalas within the region. All the koalas within the Mitchell Grass Downs (where the drainage lines were usually dry), and those koalas without access to riparian habitat within the Brigalow Belt South bioregion, had a farm dam either within their home range or within 1 km of its boundary. In fact, we found that one koala within the Brigalow Belt South travelled approximately 1 km from its core area to reach a farm dam. Within the Mitchell Grass Downs, koala faecal pellets were found at the water’s edge of a dam with no trees or over hanging branches close by. The conclusion drawn was that a koala had come down to the water to drink. In Gunnedah, north-western NSW, Lunney *et al.*[44] found that in heatwaves during a drought, about 25% of the koala population perished from dehydration. This provides further evidence that koalas utilize dams as water sources during severe droughts and heatwaves. Further, the presence of freestanding water resulted in smaller home range sizes. Therefore, the availability of water is an important component of habitat quality that influences spatial variation of home ranges.

### Approach and limitations

We used direct monitoring of individuals to investigate home range size and resource use rather than indirect methods (pellet surveys). This resulted in smaller sample sizes compared with indirect methods, but it enabled us to identify individual resource use preferences that would be unobtainable by indirect methods. Direct monitoring also revealed the importance of freestanding water and rainfall on the spatial distribution of koalas.

Equipment failures and other technical problems delayed the fieldwork and four koalas had insufficient data to warrant inclusion. This limitation in the use of GPS-tracking were not peculiar to this study [45]. To increase the sample size, three koalas whose home ranges approached, but did not reach, the asymptote were included in analyses. The collars worn by these three koalas malfunctioned and did not take all of the programmed fixes. However, the last set of points on the asymptote graphs of two of these koalas were lower than the peak, indicating that their home ranges were close to leveling off. A number of other koalas were observed either within or close to the boundaries of the home ranges of these three koalas, so it is reasonable to assume that these home ranges would not have expanded much beyond that observed. In addition, 71% of the koalas reached an asymptote between 100-200 fixes, suggesting that the minimum number of fixes required to provide reliable home range estimates is over 100 fixes and these three koalas satisfied this criterion.

## Conclusions

This study advances our understanding of the movement ecology and resource selection of an arboreal marsupial in highly dynamic, semi-arid environments such as semi-arid Australia. It also highlights the high habitat selectivity and the importance of riparian habitats for koalas living at the semi-arid edge of their distribution. Results also highlight that, within a semi-arid landscape, both rainfall (long- and short-term) and the availability of freestanding water are the primary drivers of koala home range size. Riparian habitats are critical for the long-term conservation of koala populations in semi-arid western Queensland. Historical land management practices have diminished koala habitat along drainage lines due to the silting of previously-permanent water-holes [22]. Conservation efforts within the semi-arid lands should strive to minimize further degradation of riparian habitats, as well as to maintain the quality and quantity of riparian habitats, water availability (including dams), food and shelter resources. The difference in movement patterns and resource use within the different koala populations, in response to rainfall and water availability, shows that we cannot rely upon behavioral traits of animals located towards the core of their geographic range to make assumptions about the movements and resource selection at the semi-arid edge of their range. Therefore, for conservation actions to be effective, it is imperative to distinguish differences between edge and core populations, particularly for threatened species such as the koala.

## Methods

### Study areas

**Figure 7:**
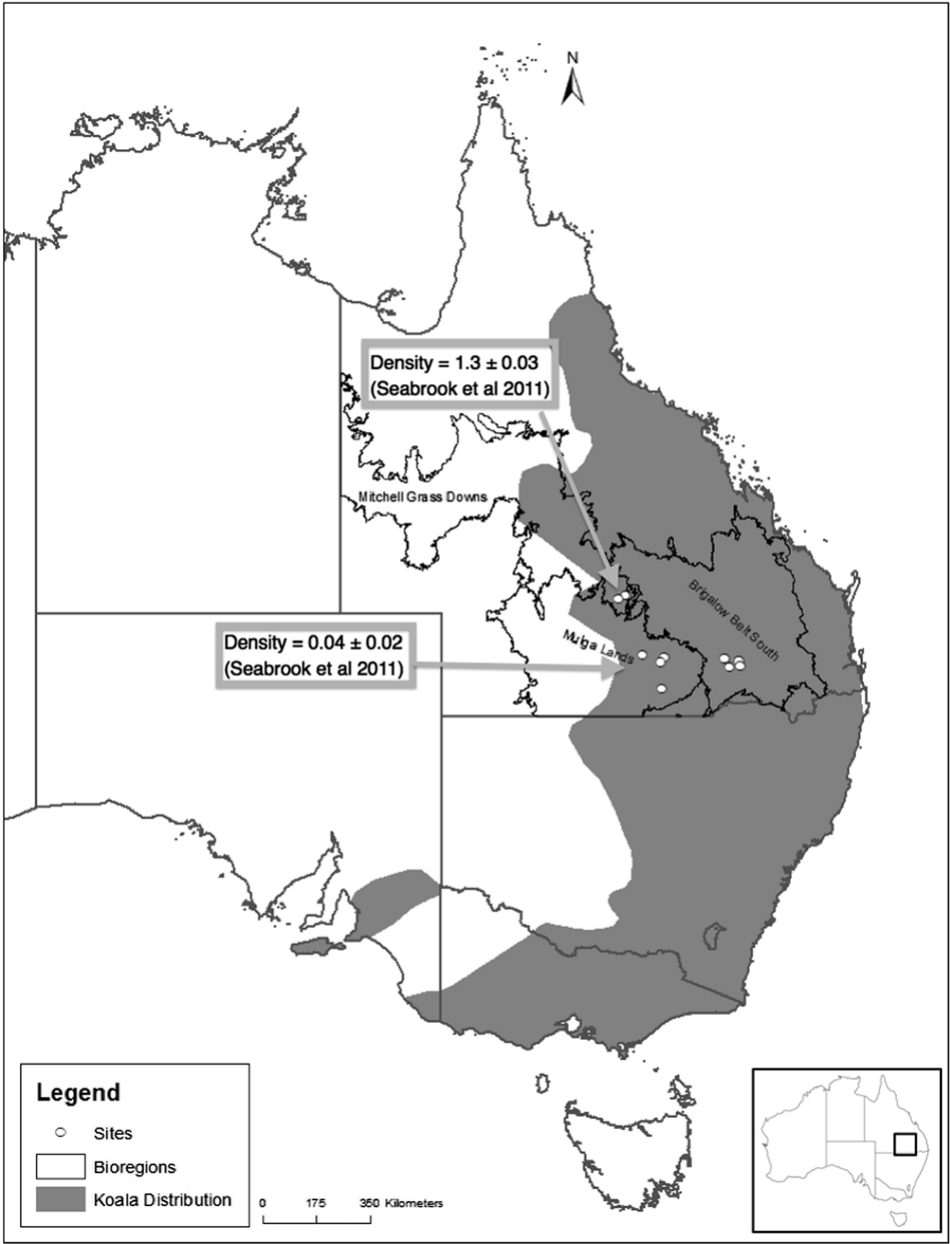
**Study area.** Southwest Queensland study area within the Mulga Lands, Mitchell Grass Downs and Brigalow Belt South bioregions. White dots show the site locations within each bioregion where koalas were collared. Map also shows where the study sites are located in relation to the geographic distribution of koalas. (Source: modified from [23, 47]).

This study was conducted at the western edge of the koala’s distribution in semi-arid, southwestern Queensland. It comprised portions of the Mulga Lands bioregion (eastern portion), the Mitchell Grass Downs bioregion (southeastern corner) and the Brigalow Belt South bioregion (western portion) (Figure 7). Annual average rainfall ranges from 750 mm in the east declining to 250 mm in the west. Rain falls mainly in summer and is highly variable [46].

Southwestern Queensland is a semi-arid landscape with a highly variable climate (Additional file 4: images of the landscapes characteristic of the study areas). Climate change predictions in this region are that the intensity of droughts and heat waves will increase and become more frequent and moisture availability will decrease [48–50].

The Mulga Lands bioregion is dominated by flat to undulating plains and low ranges supporting *Acacia aneura* (mulga) shrubland and low woodlands [51]. The woodlands that dominate waterways and associated floodplains are predominantly comprised of *Acacia* spp., *Eucalyptus populnea* (poplar box), *E. camaldulensis*, *E. coolabah* (coolabah) and *E. orchophloia* (yapunyah) [51]. Mean summer temperatures range from 21°C to 35°C, with mean winter temperatures of 5°C to 19°C [46].

The western portion of the Brigalow Belt South bioregion is predominantly comprised of *Acacia harpophylla* (brigalow), *Casuarina cristata* (belah) and *E. populnea* open-forest woodland [51]. Riparian vegetation is dominated by *E. camaldulensis*, *E. coolabah* and *E. largiflorens* (black box) [52]. The mean summer temperature within the western portion of the Brigalow Belt South ranges from 20.4°C to 34.4°C, while the mean winter temperatures are 4°C to 19°C [46].

The Mitchell Grass Downs bioregion is dominated by treeless plains of *Astrebla spp.* (Mitchell grass) with some occasional ridges, rivers and gorges. Patches of low open woodland of *E. coolabah* occur across the region in association with low-lying plains and drainage lines [52]. A drainage line is a category of watercourse that does not have a clearly defined bed or bank and only carries water during or immediately after heavy rainfall [53]. Mean summer temperatures range from 19°C to 35°C, and the mean winter temperatures range from 3.5°C to 19°C [46].

### Koala capture and tracking

Koalas were collared in each of the three different bioregions (Figure 7). Throughout the study area, 21 adult koalas were captured using the flag and pole technique [41]. Captured koalas were fitted with telemetry collars fitted with GPS units (koala modified versions of GPS data logger collars, Titley Scientific Australia and MiniTrack collars, Lotek, Wireless Inc., Canada). Both types of collars weighed < 300 g, (adult koala body weight was in the 4-9 kg range). The GPS was set to record a position six times in 24 hours (Times: Dawn - Dusk (Day) 06:00, 10:00, 14:00; Dusk – Dawn (Night) 18:00, 22:00, 02:00). Tracking was conducted from August 2010 to November 2011. Each koala was collared for 3-5 months, which allowed for movement patterns and resource preference patterns to be determined under a range of temperature and rainfall conditions. GPS units allow far more location data to be collected, especially night readings, which are hard to collect using VHF methods, and could not be collected from all koalas simultaneously.

### Home range determination and sampling

The geo-referenced data from the GPS units (mean HDOP ± SD was 1.6 ± 1.1) were used to calculate the home range sizes in ArcMap (ArcGIS 10, ESRI, Australia) using the telemetry extension package ABODE [54]. Each position was mapped in ArcMap 10, which provided a visual indication of the home ranges. Home range sizes were calculated from the 95% fixed kernel (FK) distributions. The fixed kernel can emphasize the areas of greatest use while not being highly sensitive to outliers [55]. Fixed kernel core areas were also calculated using the ‘core’ option in ABODE [54]. As recommended, *post hoc* visual assessment of plots containing the areas and probabilities for each analysis was conducted [54]. Minimum convex polygons (MCP) were also calculated to allow comparisons among other koala home range studies (Additional file 1) [56]. To ensure that the sampling duration covered the full range of each koala, home range asymptotes were estimated, using ABODE [56].

The straight-line distance between each consecutive location was measured using Geospatial Modeling Environment [57]. Diurnal and nocturnal distances travelled were estimated for each animal as the gross sum of the straight-line distances between consecutive locations per day (Dawn–Dusk: 06:00-18:00) and night (Dusk-Dawn: 18:00-06:00) period.

Where necessary, data were transformed to satisfy assumption of normal distribution and homogeneity of variances for parametric tests. Where assumptions of parametric tests could not be satisfied, non-parametric equivalents were used. We tested for variations in home range size and travel distances by bioregion and sex using one-way ANOVA (or Kruskal-Wallis test where parametric assumptions could not be met) and t-tests.

### Fractal analysis

Fractal dimension (D) gives a measure of tortuosity, or crookedness, and can provide a good quantitative description of animal movement patterns and the relative importance of environmental and behavioral factors influencing movement [58, 59]. The fractal D for movement paths lies between 1 and 2 (i.e. D is 1 when the path is straight and a maximum of 2 when the path is so tortuous as to completely cover a plane/area) [60]. Straighter movement paths may be a result of animals searching for dispersed resources such as mates or forage during low forage availability [61, 62], while more tortuous paths may indicate an area where an animal is foraging more intensively [59, 63–68]. However, Fractal D is scale-dependent, with D being lesser or greater when the path is viewed at different spatial scales [69]. Traversing the home range, searching for resources or dispersing to new habitats are likely to be very different types of movement conducted in different “domains” of scale [64, 70]. It is considered that a change in D with spatial scale signifies a transition between domains, allowing the interpretation to be made that the animal changes the way in which it interacts with its environment at that scale [59, 64–68]. Therefore, to determine the scales koalas are viewing their habitat/environment, it is important to measure not only the overall fractal D but also to measure how fractal D changes with scale [66]. We used both the Fractal Mean and VFractal estimators using the program Fractal 5 (V. O. Nams, Nova Scotia Agricultural College, Truro, Nova Scotia, Canada). We also used VFractal Correlation of Cosine estimator, which measures correlation in tortuosity of successive path segments at various scales, to detect whether animals use a hierarchical patch structure [67]. At scales smaller than the patch, if one path segment is inside a patch then the consecutive segment is also likely to be inside the patch, and likewise for segments lying outside patches [59, 66, 67]. Correlations of tortuosity of successive path segments should be positive when path lengths are below patch size, negative at patch size, and 0 when path lengths are larger than patch size, thus patch size may be estimated as the spatial scale at which the correlation declines below zero [67].

We combined all individuals within each sex and each bioregion for the estimations. When combined, VFractal treats each movement path (i.e. 1 path/koala) as 1 replicate, allowing error estimates to be based on measures of among-path variation [66], which allowed for extrapolation to each sex or bioregion. Movement paths were also weighted by N in order to minimise the effects of parameter variability (i.e. statistics at each spatial scale are weighted by the number of sampling intervals at the scale) [69]. Fractal calculates confidence intervals by bootstrapping for the VFractal estimate, adjusting the number of replications to ensure the smaller number of turning angles at large dividers sizes does not artificially inflate variance estimates. To ensure that D would be a useful relative measure of tortuosity, it was calculated over the same range of spatial scales for all individuals. To detect patch use and determine the size of patches, the correlation in tortuosity of successive path segments were plotted against spatial scale, recording the spatial scales at which correlations dropped below zero [68].

### Resource selection analysis

Each tree utilized by a collared koala was located in the field using a hand-held GPS (Garmin Oregon 300), identified to species, and its relative condition recorded (scored on a scale of 1 [poor] to 6, based on the amount of dieback). We are confident the precise trees the koalas were using were correctly identified as the GPS fixes taken by the collars were accurate (HDOP mean ± SD was 1.6 ± 1.1 m) and there were scratches on the trunks and koala faecal pellets under most of the trees identified. Furthermore, as can be seen from the images in Additional file 4, the density of the trees in most of the habitats was sparse, allowing each tree to be easily identified. Whether the used trees were located within riparian or non-riparian habitat was also recorded. The location, species and condition of each tree made up the ‘tree use’ dataset.

Time spent in either riparian or non-riparian habitat was determined from the percentage of four hourly GPS locations inside each habitat group (riparian/non-riparian) for each koala (n = 17, with 6 koalas from the Mulga Lands, 7 koalas from the Brigalow Belt South, and 4 koalas from the Mitchell Grass Downs bioregions: mean of 429.4, SD = 225.6). An ANOVA was used to compare the average time spent in riparian habitat for each bioregion. We also noted the availability of farm dams and freestanding water at each site in relation to koala home ranges.

### Variables for the SEM and multiple regression analysis

The dependent variable was koala home range size (log home range). We also considered overall distance travelled and fractal dimension as alternative dependent variables. The independent/explanatory variables are presented in Table 4. Climate data from the Bureau of Meteorology (1990-2011) was examined for stations closest to each site. Total rainfall over the two months prior to each koala being collared was collated for each site (two-month rainfall). This variable was selected for a number of reasons. Previous research found that rainfall two months prior to sample collection had a significant negative influence on the physiological stress levels of koalas (Davies *et al.* unpublished data). Changes in environmental conditions, such as climate variability, can affect population dynamics (including dispersal ability, breeding success and mortality rates), and these changes can result in population expansion or contraction as environmental conditions fluctuate [12, 35, 36]. For koalas, where habitat quality is critical, time lags can occur between rainfall events and the subsequent response by vegetation, with previous studies showing that total rainfall of the past two months has a strong effect on vegetation growth [37–39].

**Table 4:** **Explanatory variables**

Variable	Type	Full description
Sex	Categorical	Sex of each animal
Tree condition	Scale	Average condition (scale 1 [poor] to 6, based on dieback) of the trees used by each koala – calculated from the ‘tree use’ dataset
Total Nitrogen	Continuous	Soil total nitrogen content within home ranges - derived from geology mapping of Queensland (The Queensland combined soils dataset)
Annual rainfall	Continuous	Average annual rainfall (mm) for each site
2 month rainfall	Continuous	Totalled rainfall (mm) at each site 2 months prior to each koala being collared
Temperature	Continuous	Average annual min/max temp (°C) at each site
Edge distance	Continuous	Distance (km) of each site from the western edge of the koala’s geographical range – measured in ArcGIS 10 (ESRI, Australia) using koala distribution maps
Freestanding water	Categorical	Availability of freestanding water (no water, creek water, dam water)
Bioregion	Categorical	Bioregion (Mulga Lands, Mitchell Grass Downs, Brigalow Belt South)
Basin	Categorical	Basin (Warrego, Moonie, Balonne-Condamine)
Catchment	Categorical	Catchment (Mungallala, Warrego, Condamine, Moonie)

Description of explanatory variables used to determine the influence of koala home range sizes.

### Statistical analyses

Statistical analysis was conducted using the R [71] and Stata statistical software packages [72] to determine and understand the dependences between the following variables: koala home range size (dependent variable) and sex, tree condition, freestanding water, annual rainfall, rainfall for previous two-month period, and nitrogen (the independent variables). The Shapiro-Wilk test for normality of the data showed the home range variable was not distributed normally (*p*-value < 0.001). Therefore, a new log home range variable was created by logarithmic transformation of the home range values. No statistically significant dependences of the log home range were found on bioregions, catchments, basins, or distance from the western edge of the koala’s distribution. Therefore, these independent variables were omitted from further analysis. The one-way analysis of variance with multiple comparisons was applied to the log home range to evaluate any possible relationships between this variable and the location where the data was collected.

Preliminary analysis of the data revealed that all the major variables - log home range size, tree condition, availability of freestanding water, annual rainfall, two-month rainfall, and nitrogen – displayed mutual correlations (Additional file 3). The presence of the large number of mutually correlated pairs of variables presents a problem for regression analysis. Therefore, to understand the complex mutual relationships between these variables, a structural equation model (SEM) was used [73–77]. This approach is particularly useful in the case of multiple variables with no initial knowledge of which variables are capable of influencing the other variables. SEM allows identification and quantification of possible pathways for mutual influences of the involved variables, and their direct and indirect effects. The SEM model fit was assessed using: (i) the model *p*-value; (ii) residual indices including the standardized root mean squared residual [78]; (iii) fit indices including the comparative fit index [79], Tucker-Lewis index [80], and the coefficient of determination *R*^2^.

As a result of the SEM analysis, we determined the direct and indirect effects of the independent variables (in our case, annual rainfall, two-month rainfall, sex, freestanding water, tree condition, and nitrogen) on the log home range variable. However, the SEM model is a linear model that does not take into account possible interactions between the independent variables. To investigate these relationships, we used multiple regression with the independent variables that had statistically significant impacts on the log home range variable. In this way, we determined the mutual influences and relative importance of the variables in the set and their impacts on the dependent variable (log home range). When plotting the dependences of home range versus other variables, back-transformation of the log home range variable was used, including for the 95% prediction intervals.

To further verify the constructed multiple regression model, the model averaging procedure [81] was applied to all the considered variables including the interactions between sex and annual rainfall, and between sex and two-month rainfall. The interactions with the freestanding water variable were not taken into account in the models because of strong and obvious correlations between this variable and the annual rainfall. In the model averaging procedure, the models with all possible combinations of the independent variables and their interactions were considered, and the fit for each model was evaluated using AIC [81]. The 95% confidence set of the best models was identified, and the averaged coefficients were calculated based on this confidence set.

Overall distance travelled and fractal dimension were used as alternative dependent variables (instead of log home range) in the above statistical analyses (multiple regression, model average and SEM). This was done to see how much influence the environmental variables have in explaining movement patterns. This also allowed us to consider different options for the dependent variable to find the optimal model choice that gave the best statistical outcomes.

## Availability of supporting data

The data sets supporting the results of this article are included within the article (and its additional files).

## Electronic supplementary material

Additional file 1: **HR summary table.** Collar data summary. Description: Summary of the collar data collected for each koala. * Included in home range analysis despite home range not reaching an asymptote. (PDF 93 KB)

Additional file 2: **HR maps & overlap.** Home range maps. Description: Maps showing the spatial distribution of koala home ranges within each bioregion against the availability of water sources (drainage and/or dam). Map shows the GPS fixes, the 95% FK home range contours, core areas and MCP outline for each koala. (a - d) Mitchell Grass Downs; (e – j) Mulga Lands; (k - q) Brigalow Belt South koalas. All koalas collared at a site within the (r) Mitchell Grass Downs, (s) Brigalow Belt South, and (t) Mulga Lands bioregion – maps shows the extent of overlapping between 95% FK home range contours (please note that not every koala within each site was collared so there may be other koalas living within the collared koala’s home range). (u) Map shows the core FK home range contours for a Mulga Lands site – note the core areas of the two males (Kai and Unwin) do not overlap and that they each overlap with a female core area, whilst the 95% FK contours of the same two males did overlap (t). (PDF 17 MB)

Additional file 3: **Correlated variables.** Correlated variables. Description: Simple correlations between the pairs of the major variables (only correlations with p < 0.1 are included). (PDF 68 KB)

Additional file 4: **Study area photos.** Study area photographs. Description: Photos displaying the landscapes that characterize the study areas and that highlight the relatively sparse, dry habitats with only a few eucalypt species present. (a) Non-riparian *E. populnea* (poplar box) woodland of the Mulga Lands bioregion; (b) riparian habitat dominated by *E. camaldulensis* (river red gum) within the Mulga Lands bioregion– dry creek bed; (c) riparian habitat dominated by *E. camaldulensis* within the Mulga Lands bioregion – free-standing water present; (d) riparian habitat dominated by *E. camaldulensis* within the Brigalow Belt South bioregion; (e) non-riparian *E. populnea* (poplar box) woodland of the Brigalow Belt South bioregion; (f) non-riparian *E. populnea* (poplar box) and *A. harpophylla* (brigalow) woodlands of the Brigalow Belt South bioregion; (g) drainage line habitat dominated by *E. coolabah* low open woodland within the Mitchell Grass Downs bioregion; (h) plains (non-riparian) habitat supporting a dam within the Mitchell Grass Downs bioregion. (PDF 479 KB)
