# Supplementary material for: Movement patterns of an arboreal marsupial at the edge of its range: a case study of the koala
Source: Mov Ecol. 2013 Sep 12;1(1):8. doi: 10.1186/2051-3933-1-8 (PMC4337771; doi:10.1186/2051-3933-1-8)
Supplement: Supplementary file 1 — Additional file 1: HR summary table. Collar data summary. Description: Summary of the collar data collected for each koala. * Included in home range analysis despite home range not reaching an asymptote. (PDF 93 KB) [file 40462_2013_8_MOESM1_ESM.pdf]

Bioregion	Koala	Sex	Period Tracked	Days	Fixes	MCP (ha)	FK (ha)	Core (ha)	Asymptote
Mitchell	1 (Caramella)	Female	18 Aug–20 Nov 2010	93	419	15.4	8.3	2.8	Yes
Grass	13 (Wilma)	Female	29 May – 9 Aug 2011	80	470	50.2	34.6	9.5	Yes
Downs	11 (AKW)	Male	22 May- 25 July 2011	63	348	41.2	24.4	8.1	Yes
	12 (Betty)	Female	23 May - 5 Nov 2011	154	813	19.7	15.4	4.1	Yes
Mulga	2 (Nutsy)	Female	24 Aug - 19 Dec 2010	118	570	257.5	210.5	88.3	Yes
Lands	14 (Yoda)	Male	30 May- 12 June 2011	14	66	5	7.3	1.3	No
	3 (Binga)	Female	31 Aug - 20 Dec 2010	112	618	74.7	66.7	18.4	Yes
	9 (Kai)	Male	18 May - 6 July 2011	51	248	560.9	571.5	118.6	*No
	10 (Beanie)	Female	18 May - 4 Nov 2011	171	991	201.1	105.3	27.5	Yes
	17 (Sweetie)	Female	11 Aug - 4 Nov 2011	85	390	16.3	8.1	1.8	Yes
	18 (Unwin)	Male	13 Aug- 30 Sept 2011	45	230	64.1	54.8	15.9	Yes
Brigalow	4 (Bundy Bear)	Male	3 Sept - 24 Nov 2010	64	318	39.8	19.4	8.0	Yes
Belt	5 (Honey)	Female	12 May - 18 June 2011	37	203	9.5	7.5	1.8	Yes
	5 (Blinky Bill)	Male	13 May - 3 Sept 2011	95	435	30	24.4	10.4	Yes
	8 (Sumo)	Male	15 May-6 June & 7-30 Aug 2011	47	193	77	70.3	23.4	*No
	7 (Wolverine)	Male	13 May - 2 July 2011	51	130	78.3	46	10.9	*No
	15 (Chase)	Male	3 Aug -1 Nov 2011	85	426	76.3	49.9	10.2	Yes
	16 (Sleeping Beauty)	Female	4 Aug - 3 Nov 2011	91	497	19	10.6	3.8	Yes
