# Supplementary material for: Movement patterns of an arboreal marsupial at the edge of its range: a case study of the koala
Source: Mov Ecol. 2013 Sep 12;1(1):8. doi: 10.1186/2051-3933-1-8 (PMC4337771; doi:10.1186/2051-3933-1-8)
Supplement: Supplementary file 2 — Additional file 2: HR maps & overlap. Home range maps. Description: Maps showing the spatial distribution of koala home ranges within each bioregion against the availability of water sources (drainage and/or dam). Map shows the GPS fixes, the 95% FK home range contours, core areas and MCP outline for each koala. (a - d) Mitchell Grass Downs; (e – j) Mulga Lands; (k - q) Brigalow Belt South koalas. All koalas collared at a site within the (r) Mitchell Grass Downs, (s) Brigalow Belt South, and (t) Mulga Lands bioregion – maps shows the extent of overlapping between 95% FK home range contours (please note that not every koala within each site was collared so there may be other koalas living within the collared koala’s home range). (u) Map shows the core FK home range contours for a Mulga Lands site – note the core areas of the two males (Kai and Unwin) do not overlap and that they each overlap with a female core area, whilst the 95% FK contours of the same two males did overlap (t). (PDF 17 MB) [file 40462_2013_8_MOESM2_ESM.pdf]

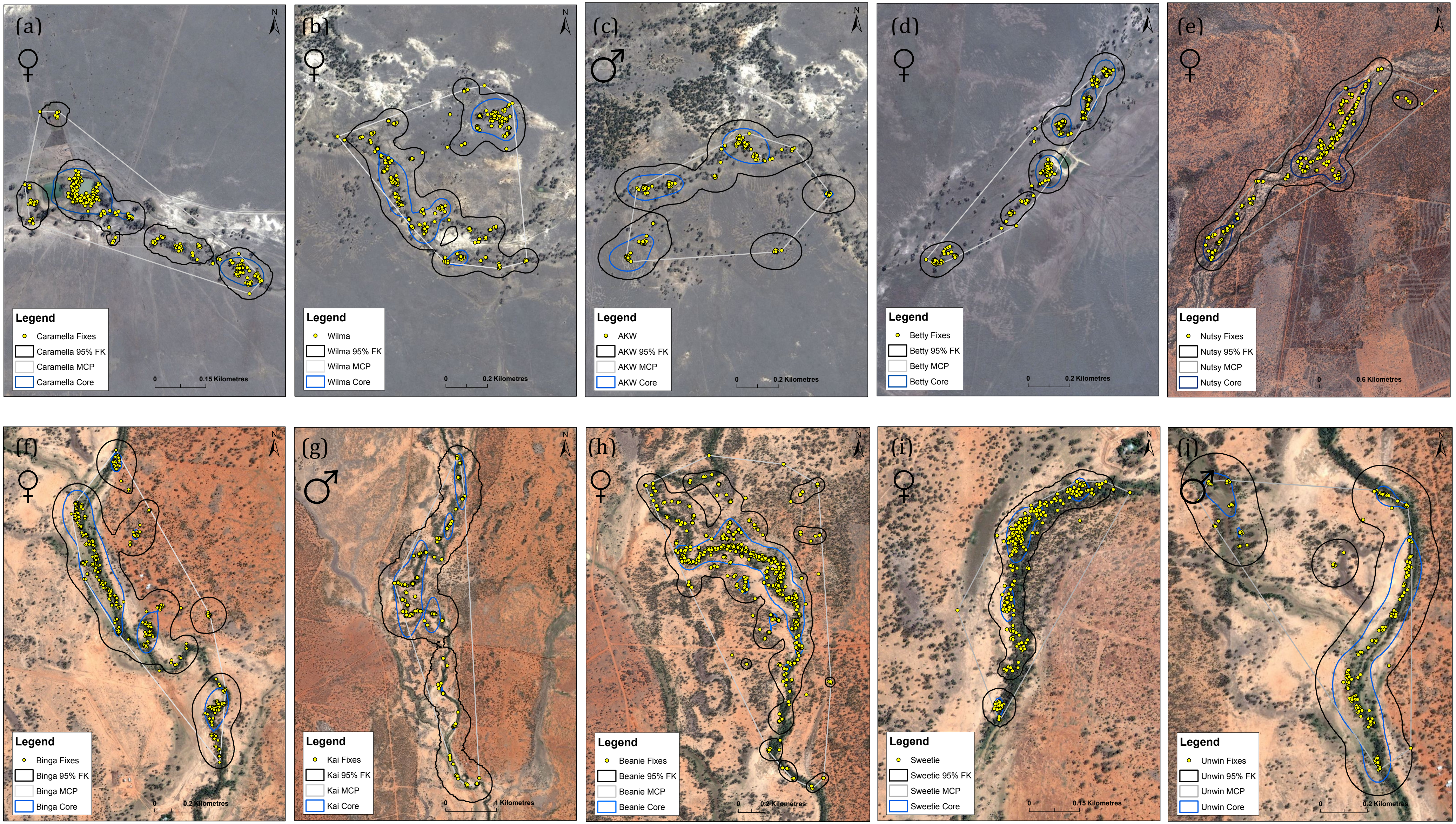
(a)
Legend
Caramella Fixes
Caramella 95% FK
Caramella MCP
Caramella Core
0 0.15 Kilometres
(b)
Legend
Wilma
Wilma 95% FK
Wilma MCP
Wilma Core
0 0.2 Kilometres
(c)
Legend
AKW
AKW 95% FK
AKW MCP
AKW Core
0 0.2 Kilometres
(d)
Legend
Betty Fixes
Betty 95% FK
Betty MCP
Betty Core
0 0.2 Kilometres
(e)
Legend
Nutsy Fixes
Nutsy 95% FK
Nutsy MCP
Nutsy Core
0 0.6 Kilometres
(f)
Legend
Binga Fixes
Binga 95% FK
Binga MCP
Binga Core
0 0.2 Kilometres
(g)
Legend
Kai Fixes
Kai 95% FK
Kai MCP
Kai Core
0 1 Kilometres
(h)
Legend
Beanie Fixes
Beanie 95% FK
Beanie MCP
Beanie Core
0 0.2 Kilometres
(i)
Legend
Sweetie
Sweetie 95% FK
Sweetie MCP
Sweetie Core
0 0.15 Kilometres
(j)
Legend
Unwin Fixes
Unwin 95% FK
Unwin MCP
Unwin Core
0 0.2 Kilometres

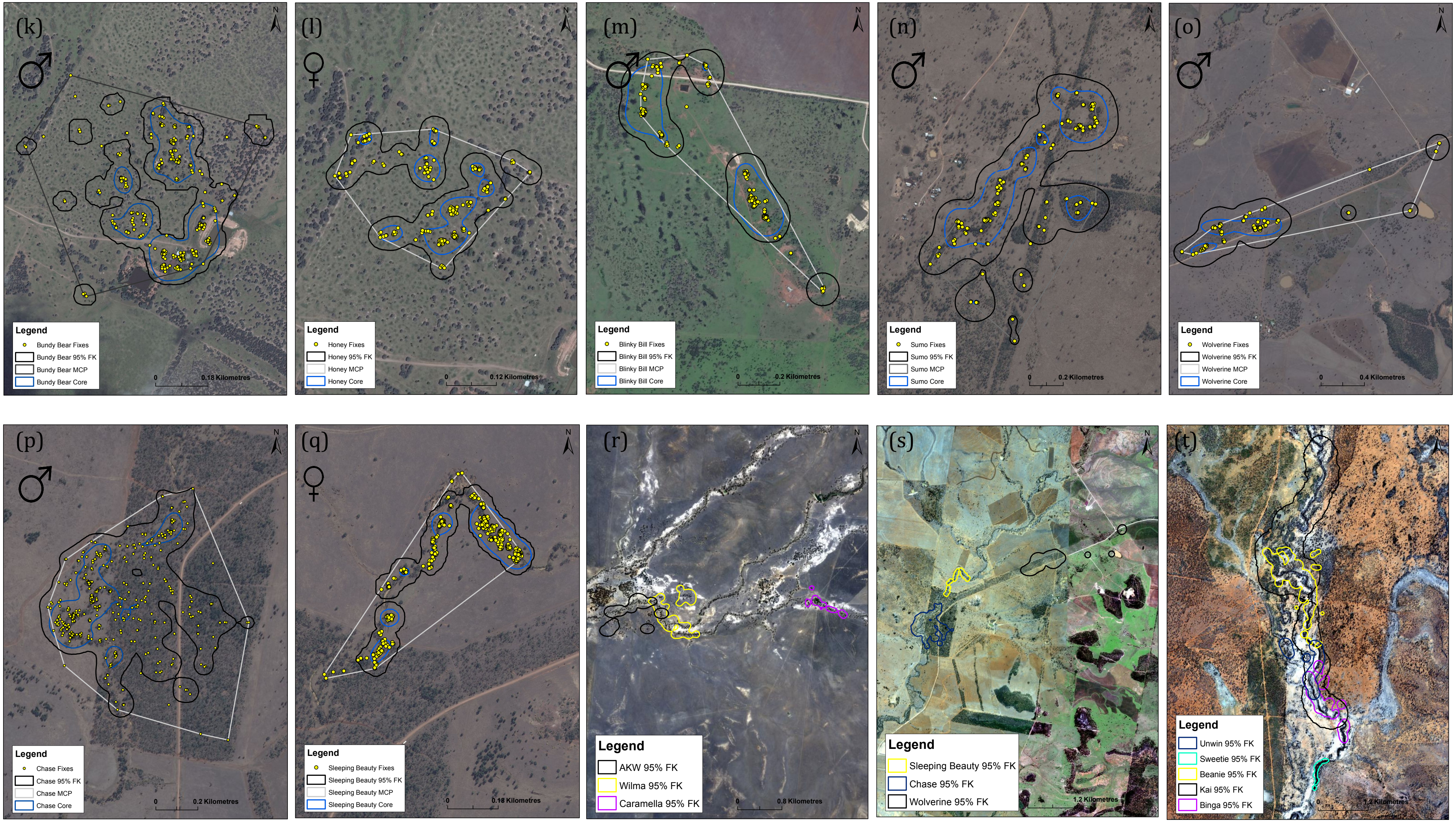
(k)
Legend
Bundy Bear Fixes
Bundy Bear 95% FK
Bundy Bear MCP
Bundy Bear Core
0 0.18 Kilometres
(l)
Legend
Honey Fixes
Honey 95% FK
Honey MCP
Honey Core
0 0.12 Kilometres
(m)
Legend
Blinky Bill Fixes
Blinky Bill 95% FK
Blinky Bill MCP
Blinky Bill Core
0 0.2 Kilometres
(n)
Legend
Sumo Fixes
Sumo 95% FK
Sumo MCP
Sumo Core
0 0.2 Kilometres
(o)
Legend
Wolverine Fixes
Wolverine 95% FK
Wolverine MCP
Wolverine Core
0 0.4 Kilometres
(p)
Legend
Chase Fixes
Chase 95% FK
Chase MCP
Chase Core
0 0.2 Kilometres
(q)
Legend
Sleeping Beauty Fixes
Sleeping Beauty 95% FK
Sleeping Beauty MCP
Sleeping Beauty Core
0 0.18 Kilometres
(r)
Legend
AKW 95% FK
Wilma 95% FK
Caramella 95% FK
0 0.8 Kilometres
(s)
Legend
Sleeping Beauty 95% FK
Chase 95% FK
Wolverine 95% FK
1.2 Kilometres
(t)
Legend
Unwin 95% FK
Sweetie 95% FK
Beanie 95% FK
Kai 95% FK
Binga 95% FK
0 1.2 Kilometres

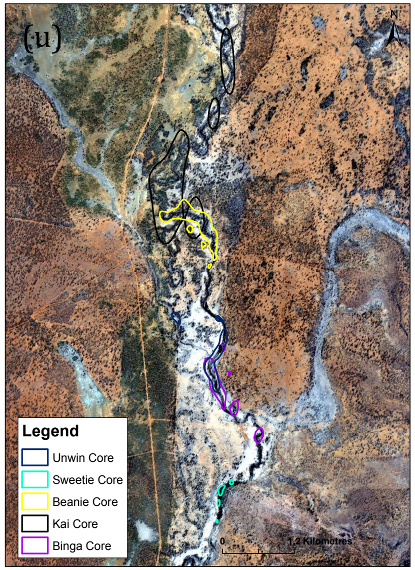

Figure B: Maps showing the spatial distribution of koala home ranges within each bioregion against the availability of water sources (drainage and/or dam). Map shows the GPS fixes, the 95% FK home range contours, core areas and MCP outline for each koala. (a - d) Mitchell Grass Downs; (e – j) Mulga Lands; (k - q) Brigalow Belt South koalas. All koalas collared at a site within the (r) Mitchell Grass Downs, (s) Brigalow Belt South, and (t) Mulga Lands bioregion – maps shows the extent of overlapping between 95% FK home range contours (please note that not every koala within each site was collared so there may be other koalas living within the collared koala's home range). (u) Map shows the core FK home range contours for a Mulga Lands site – note the core areas of the two males (Kai and Unwin) do not overlap and that they each overlap with a female core area, whilst the 95% FK contours of the same two males did overlap (t).
