# Supplementary material for: Movement patterns of an arboreal marsupial at the edge of its range: a case study of the koala
Source: Mov Ecol. 2013 Sep 12;1(1):8. doi: 10.1186/2051-3933-1-8 (PMC4337771; doi:10.1186/2051-3933-1-8)
Supplement: Supplementary file 3 — Additional file 3: Correlated variables. Correlated variables. Description: Simple correlations between the pairs of the major variables (only correlations with p < 0.1 are included). (PDF 68 KB) [file 40462_2013_8_MOESM3_ESM.pdf]

**Correlated Variables**		**Correlation Coefficient**	**p-value**
Log home range	Annual rain	-0.47	0.055
	Rain for two-month	0.47	0.056
	Freestanding water	-0.68	0.003
	Tree condition	-0.54	0.026
Annual rain	Freestanding water	0.54	0.024
	Tree condition	0.73	< 0.001
Tree condition and nitrogen		0.48	0.049
