# Supplementary material for: Movement patterns of an arboreal marsupial at the edge of its range: a case study of the koala
Source: Mov Ecol. 2013 Sep 12;1(1):8. doi: 10.1186/2051-3933-1-8 (PMC4337771; doi:10.1186/2051-3933-1-8)
Supplement: Supplementary file 4 — Additional file 4: Study area photos. Study area photographs. Description: Photos displaying the landscapes that characterize the study areas and that highlight the relatively sparse, dry habitats with only a few eucalypt species present. (a) Non-riparian E. populnea (poplar box) woodland of the Mulga Lands bioregion; (b) riparian habitat dominated by E. camaldulensis (river red gum) within the Mulga Lands bioregion– dry creek bed; (c) riparian habitat dominated by E. camaldulensis within the Mulga Lands bioregion – free-standing water present; (d) riparian habitat dominated by E. camaldulensis within the Brigalow Belt South bioregion; (e) non-riparian E. populnea (poplar box) woodland of the Brigalow Belt South bioregion; (f) non-riparian E. populnea (poplar box) and A. harpophylla (brigalow) woodlands of the Brigalow Belt South bioregion; (g) drainage line habitat dominated by E. coolabah low open woodland within the Mitchell Grass Downs bioregion; (h) plains (non-riparian) habitat supporting a dam within the Mitchell Grass Downs bioregion. (PDF 479 KB) [file 40462_2013_8_MOESM4_ESM.pdf]

(a)
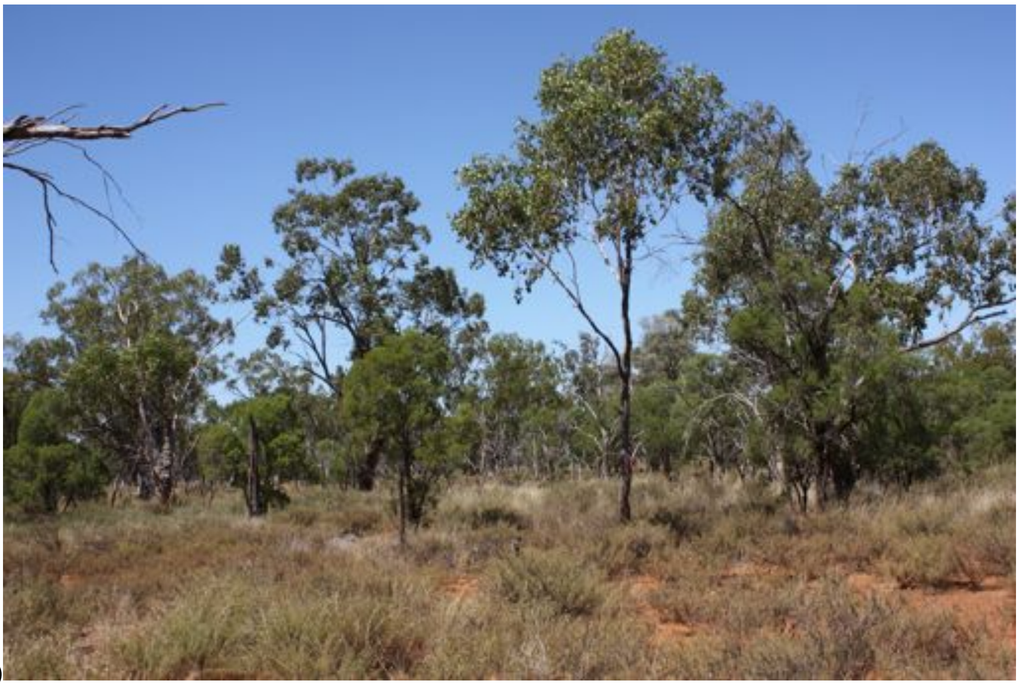
(b)
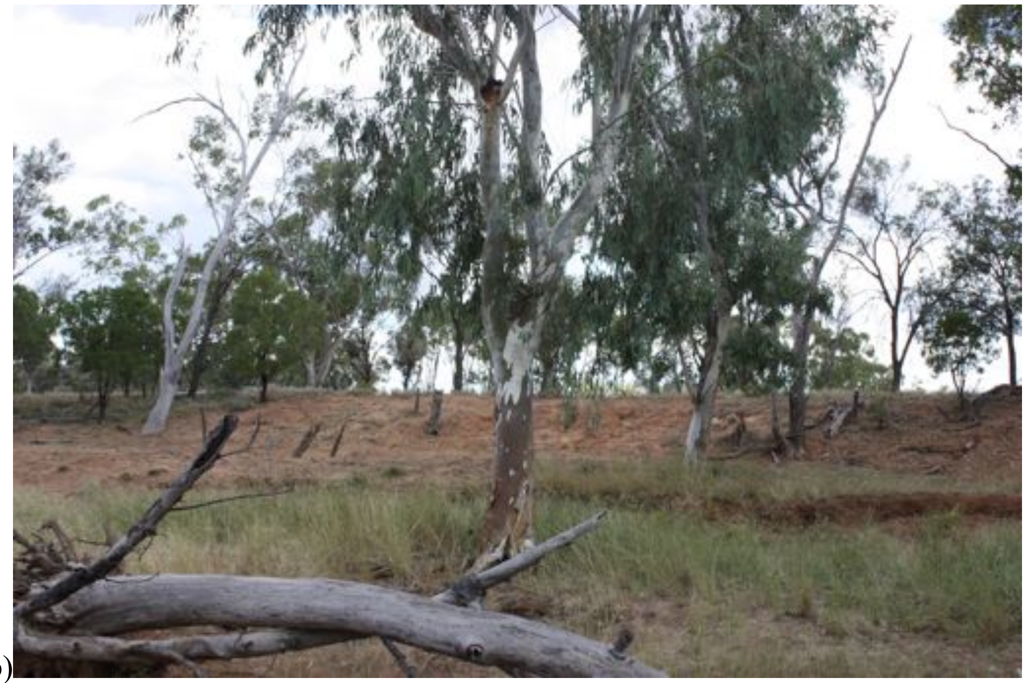
(c)
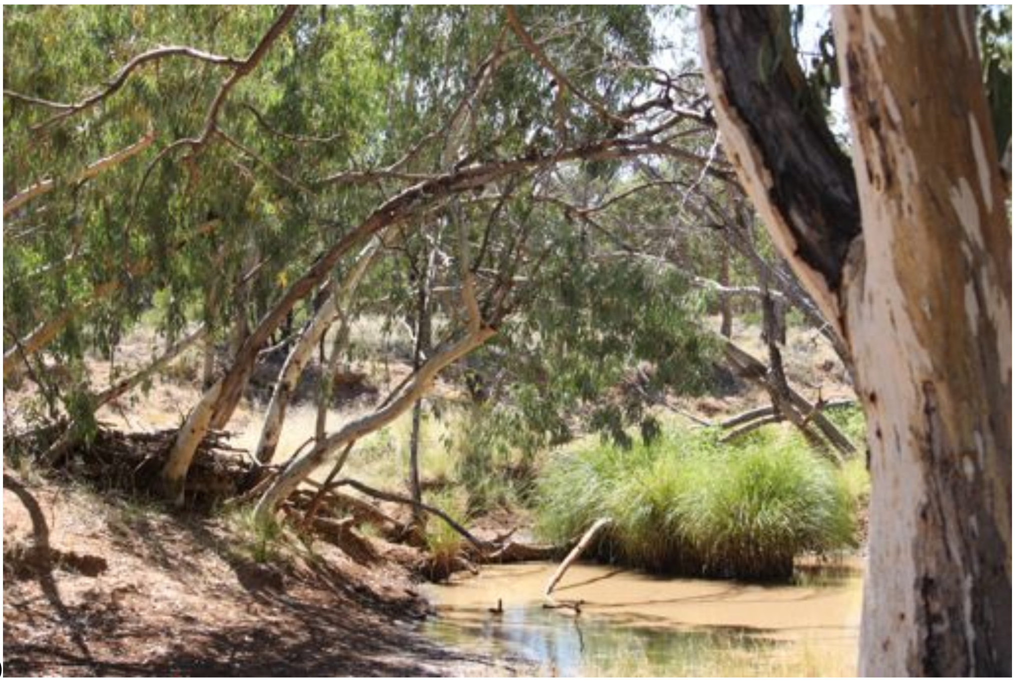

(d) 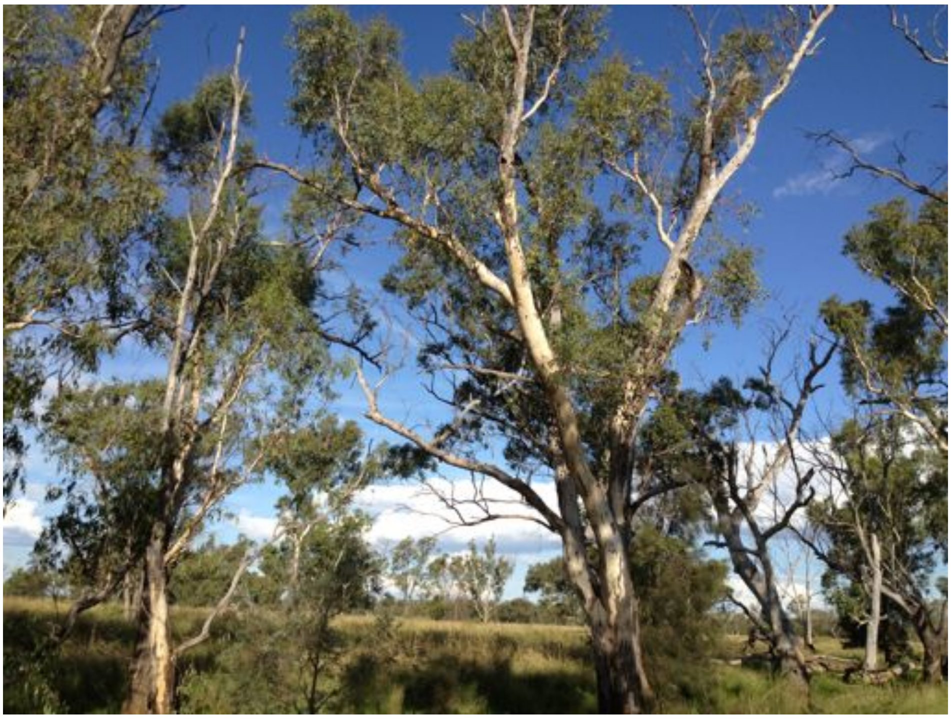

(e) 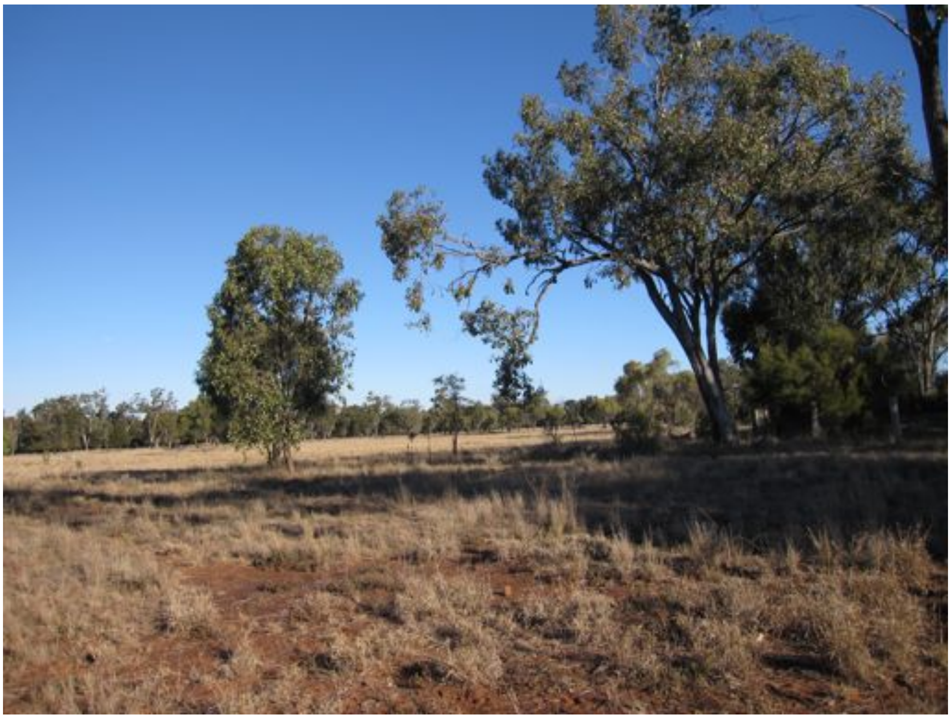

(f) 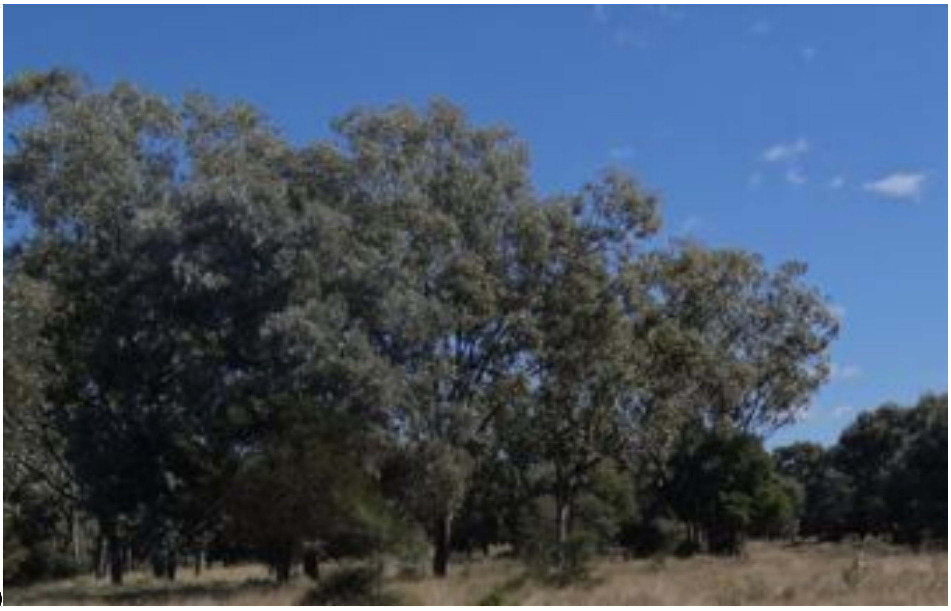

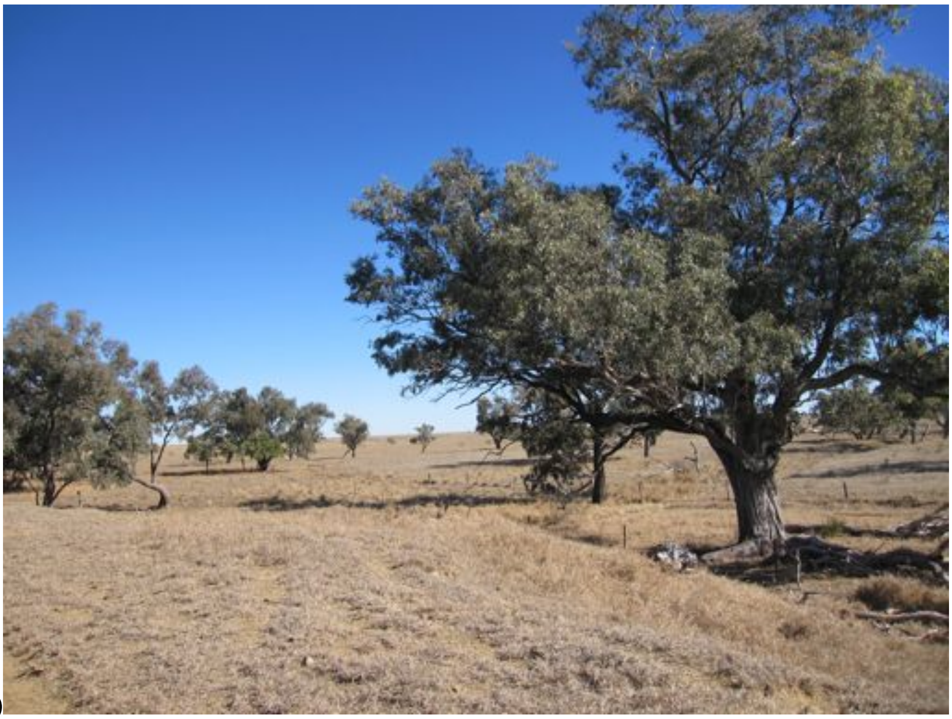

(g)

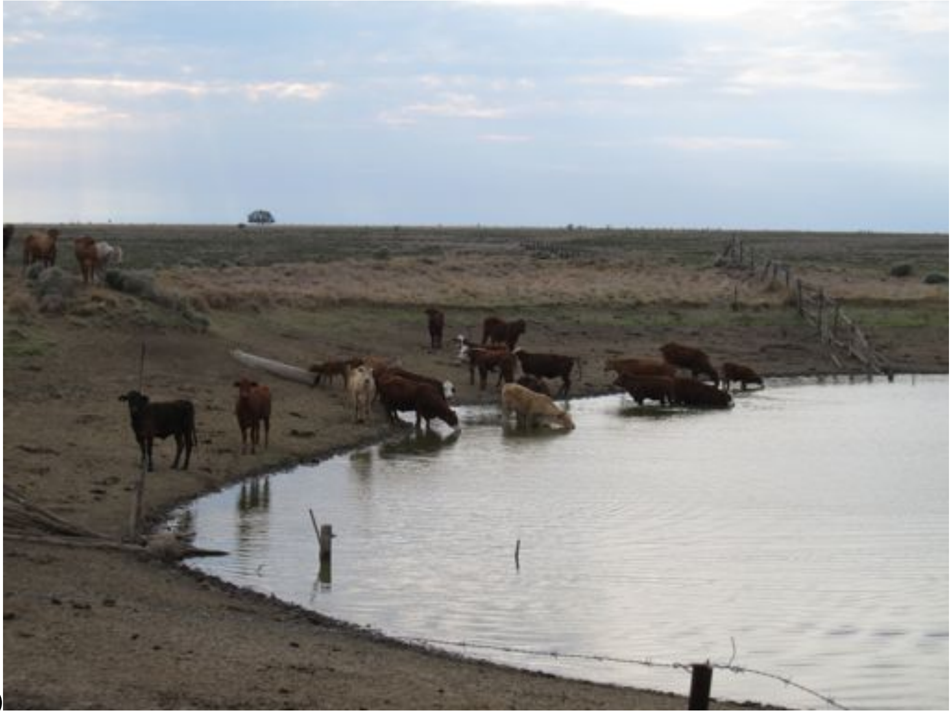

(h)

Figure A: Photos displaying the landscapes that characterize the study areas and that highlight the relatively sparse, dry habitats with only a few eucalypt species present. (a) Non-riparian *E. populnea* (poplar box) woodland of the Mulga Lands bioregion; (b) riparian habitat dominated by *E. camaldulensis* (river red gum) within the Mulga Lands bioregion– dry creek bed; (c) riparian habitat dominated by *E. camaldulensis* within the Mulga Lands bioregion – free-standing water present; (d) riparian habitat dominated by *E. camaldulensis* within the Brigalow Belt South bioregion; (e) non-riparian *E. populnea* (poplar box) woodland of the Brigalow Belt South bioregion; (f) non-riparian *E. populnea* (poplar box) and *A. harpophylla* (brigalow) woodlands of the Brigalow Belt South bioregion; (g) drainage line habitat dominated by *E. coolabah* low open woodland within the Mitchell Grass Downs bioregion; (h) plains (non-riparian) habitat supporting a dam within the Mitchell Grass Downs bioregion.
